# Thermo-Mechanical and Delamination Properties in Drilling GFRP Composites by Various Drill Angles

**DOI:** 10.3390/polym13111884

**Published:** 2021-06-06

**Authors:** Usama A. Khashaba, Mohamed S. Abd-Elwahed, Mohamed A. Eltaher, Ismail Najjar, Ammar Melaibari, Khaled I. Ahmed

**Affiliations:** 1 Mechanical Engineering Department, Faculty of Engineering, King Abdulaziz University, P.O. Box 80204, Jeddah 22254-2265, Saudi Arabia; khashabu@zu.edu.eg (U.A.K.); msabdelwahed@gmail.com (M.S.A.-E.); inajjar@kau.edu.sa (I.N.); aamelaibari@kau.edu.sa (A.M.); kahmed@kau.edu.sa (K.I.A.); 2 Mechanical Design and Production Engineering Department, Faculty of Engineering, Zagazig University, Zagazig 44519, Egypt

**Keywords:** drilling point angle, thermal and mechanical analysis, delamination assessments, regression models and optimization, woven glass fiber composites, drilling of composite

## Abstract

This manuscript aims to study the effects of drilling factors on the thermal-mechanical properties and delamination experimentally during the drilling of glass fiber reinforced polymer (GFRP). Drilling studies were carried out using a CNC machine under dry cutting conditions by 6 mm diameter with different point angles of ∅ = 100°, 118°, and 140°. The drill spindle speed (400, 800, 1600 rpm), feed (0.025, 0.05, 0.1, 0.2 mm/r), and sample thickness (2.6, 5.3, and 7.7 mm) are considered in the analysis. Heat affected zone (HAZ) generated by drilling was measured using a thermal infrared camera and two K-thermocouples installed in the internal coolant holes of the drill. Therefore, two setups were used; the first is with a rotating drill and fixed specimen holder, and the second is with a rotating holder and fixed drill bit. To measure thrust force/torque through drilling, the Kistler dynamometer model 9272 was utilized. Pull-in and push-out delamination were evaluated based on the image analyzed by an AutoCAD technique. The regression models and multivariable regression analysis were developed to find relations between the drilling factors and responses. The results illustrate the significant relations between drilling factors and drilling responses such as thrust force, delamination, and heat affect zone. It was observed that the thrust force is more inspired by feed; however, the speed effect is more trivial and marginal on the thrust force. All machining parameters have a significant effect on the measured temperature, and the largest contribution is of the laminate thickness (33.14%), followed by speed and feed (29.00% and 15.10%, respectively), ended by the lowest contribution of the drill point angle (11.85%).

## 1. Introduction

Nowadays, fiber-reinforced polymer (FRP) composites are used rather than conventional materials in many disciplines and applications because of their attractive characteristics such as excellent chemical resistance, lightweight, high stiffness, and specific strength, Abhishek et al. [1]. FRP composites are commonly used in a vast wide range of applications. These applications are aerospace and aeronautical industries, automotive, marine, ship structures, railway, pressure vessels, integrated circuits, optical fibers, pho-to active materials, and wind energy [2,3]. Machining of composite, especially drilling, is considered one of the essential operations to assemble different components in industries, needs machining with perfect hole quality and quick cycle time. Composite drilling differs from metal drilling because of fiber breakage and debonding, microcracking, matrix burning, delamination, and deterioration of the surface integrity [4,5].

Till now, the drilling of composite is still questionable for the industry and researchers. Rawat and Attia [6] used machinability maps to present the impact of drilling param-eters of CFRP on the delamination, surface roughness, and hole circularity. Palanikumar [7] optimized drilling factors, including multiple regression of thrust force, workpiece surface roughness, and delamination. Rajmohan and Palanikumar [8] predicted by response surface and central composite design that the optimized drilling parameters are minimum thrust force of 84 N and the burr height of 0.16 mm. Nasir et al. [9] evaluated a reduction in tensile strength and delamination damage of flax fiber-reinforced composites during the drilling process experimentally. Abhishek et al. [1] presented a regression model using a harmony search (HS) algorithm to evaluate performance characteristics in the drilling of carbon fiber reinforced polymer (CFRP) using a carbide drill bit. Khashaba and El-Keran [10] investigated experimentally and analytically the drilling of GFRP, showing the impact of machining parameters on drilling thrust force and delamination. Ramesh et al. [11] studied the influence of special geometry drills and self-excited vibration of the work material to reduce the delamination damage in thick composites. Qiu et al. [12] studied the influences of chisel edge and step drill structure on delamination of CFRP during the drilling process and showed that the chisel edge has a serious impact on delamination when the different ratio of primary drill bit diameter to secondary drill bit diameter (k) is bigger than 0.75. Fu et al. [13] explored drill-exit temperature characteristics in drilling UD and MD CFRPs using a microscopy infrared imaging system. Xu and Zhang [14] investigated the heat effect on the material removal mechanisms in the machining of FRP composites with/without ultrasonic tool vibration. Giasin et al. [15] studied the influences of machining parameters and cutting tool coating on hole quality in the drilling of fiber metal laminates. Formisano et al. [16] presented impacts of the manufacturing process on the mechanical properties of GFRP composite laminates. Xu et al. [17] inspected the drilling forces/temperatures and wear signatures of tools during drilling of multilayer CFRP/Ti6Al4V stacks using uncoated carbide and diamond-coated twist drills under different drilling sequence strategies. Jai et al. [18,19] presented an analytical study of delamination damage and delamination-free drilling method of CFRP composite. Erturk et al. [20] studied the effects of cutting temperature and drilling parameters (drill bits, feed rate, and spindle speed) on the delamination of GFRP composites. Galińska et al. [21] presented a comprehensive review of FRP composites’ mechanical joining using bolted joints. Rahmé et al. [22] proved, experimentally, that adding a woven glass ply at the exit of the hole of CFRP laminates is an adequate solution to reduce the defect of delamination during drilling. Khashaba et al. [23] explored experimentally thrust force, torque, and de-lamination of GFRP composites during drilling processes with different machining pa-rameters. Sobri et al. [24] developed a new methodology to evaluate the delamination fac-tor of drilled holes of thick CFRP composites. Pie et al. [25] developed a practicable and environmentally friendly strategy to recycle carbon fibers from waste CFRPs by an electrochemical catalytic reaction with the assistance of phosphotungstic acid. They also presented the existing CFRP recovery methods such as mechanical recovery, pyrolysis, fluidized bed method, and super/subcritical fluid decomposition. Li et al. [26] presented the effect of chopped carbon fiber (CF) on CF-reinforced cementitious matrix system interfacial behaviors. Zhang et al. [27,28] analyzed the impacts of axial force and hole-exit temperature on the hole-exit surface damages. Bai et al. [29] and Wang et al. [30] proposed a novel mechanical model to predict a drilling thrust force with tool wear effects of unidirectional CFRP and CFRP/Al stack.

The drill point angle is considered the most significant parameter for cutting forces and the delamination damage [31]. Ill-designed cutting-edge results in undesired distribution of the cutting angles through drill cutting edge, which may cause inefficient quality, a deficiency of cutting ability, a higher thrust force, increased push-out delamination damage, and a rise in total manufacturing cost [32,33]. Gaitonde et al. [34] emphasized that the delamination factor can be reduced up to 45% by using a drill bit with an 85°-point angle. Durao et al. [35] examined the effects of five drill geometries on thrust force and delamination and concluded that the thrust force is smaller in Dagger and Step drills than others, and the minimum delamination is observed in the case of the twist 120° drill. Kilickap [36] predicted that by increasing the point angle of the HSS drill, delamination decreased effectively during conventional drilling of UD -GFRP laminate. Ismail et al. [37] predicted, analytically, that by increasing the chisel edge ratio, the critical thrust force, and the feed rate increase. Díaz-Álvarez et al. [38] showed numerically and experimentally that higher point angles induce higher values of thrust force and, on the contrary, reduce the damage generated during the machining process. Arrospide et al. [39] examined the influence of different drilling bits on the quality of holes, surface rugosity, diameter deviation, and coaxial. Qiu et al. [40] used a compound drill bit design (dragger drill, double pint angle drill, and candlestick drill) to reduce and eliminate the ex-it-delamination during drilling CFRP. Bayraktar and Turgut [4] presented the influence of drill point angle on delamination of C/C/Al 6013-T651 stacks with uncoated and coated drills and concluded that uncoated drill has better performance than coated drill according to delamination criterion. Liu et al. [41] proved that the thrust force produced by extrusion of the chisel edge is greater than that generated by cutting the chisel edge. Shu et al. [42] conducted a comprehensive comparison between the dedicated and conventional drill bit designs from various views, such as thermal, mechanical, and chip formation.

The investigation of thermal and mechanical behaviors of the woven GRP composite laminated under different drilling bits has not to be addressed comprehensively to the author’s knowledge and the literature. Therefore, the current article aims to cover this point. The analysis of heat-affected zone (HAZ) generated during drilling by different drill bits and machining parameters are illustrated using IR thermal camera and thermocouple. The impact of these parameters on the thrust force, torque, and delamination has been evaluated. The coupling effect of machining parameters and HAZ on the critical thrust force and damage delamination has been discussed. The optimization technique using multiple regression has been used to predict the optimum machining parameters for a specific constraint. The rest of the manuscript is organized as follows; the experimental works, including specimen preparation, specimen characterization, drilling setup, and valuation of delamination, are presented in Section 2. The evolution of temperature and heat-affected zone through the drilling process are discussed and analyzed in Section 3. Results including the influence of drilling parameters such as point angle, speed, feed, and specimen thickness on the mechanical are discussed in detail through Section 4. The statistical analysis, surface plot, and optimization techniques to obtain the optimum thrust force, delamination, and generated heat are presented and discussed in Section 5. The conclusion and outcomes are summarized in Section 6.

## 2. Materials and Methods

### 2.1. Specimen Preparation and Characterization

The hand lay-up technique was exploited to fabricate the woven GFRP composite laminates with three different thicknesses of 2.6, 5.3, and 7.7 mm, constructed from 8, 16, and 24 glass fiber layers, in our lab at King Abdulaziz University (Jeddah, Saudi Arabia). The Araldite LY5138-2 epoxy polymer and HY5138 Hardener were used in the fabrication process. The fiber volume fractions of the fabricated GFRP laminates for the three samples is about 40%, where the areal weight of the fabric Aw = 324 g/m^2^, and the fiber density 2.5 g/cm^3^.

CNC abrasive waterjet machine was used to cut the tested specimens in standard dimension rather than conventional machining processes to reduce the heat generated during cutting. A series of standard tensile tests, with a rate of 1.0 mm/min, were performed according to standard ASTM D 3039 to characterize and evaluate mechanical properties of fabricated materials using Servohydraulic Instron 8803 and 8872 testing machines with capacities 500 kN and 10 kN, respectively. 4-channels data acquisition (DAQ) model 9237 NI was used to measure the longitudinal and transverse strains. For each test, five samples were evaluated, and the average value was recorded as in Table 1.

### 2.2. Experimental Procedure

CNC milling machine model “Deckel Maho DMG DMC 1035 V, ecoline” was used to perform drilling tests under dry cutting conditions. Two flute-twist drills manufactured from special ultra-fine cemented carbide particles were utilized for efficient cutting, with excellent toughness and abrasion resistance. As provided by the manufacturer (Zhuzhou Best for Tools Co., Ltd., Zhuzhou, China), the details about drill materials and geometry were illustrated in Table 2 and Table 3, respectively. Drills were provided with two internal coolant holes of 0.6 mm diameter. Dills with three different point angles 100°, 118°, and 140° were used throughout this article.

**Figure 1:**
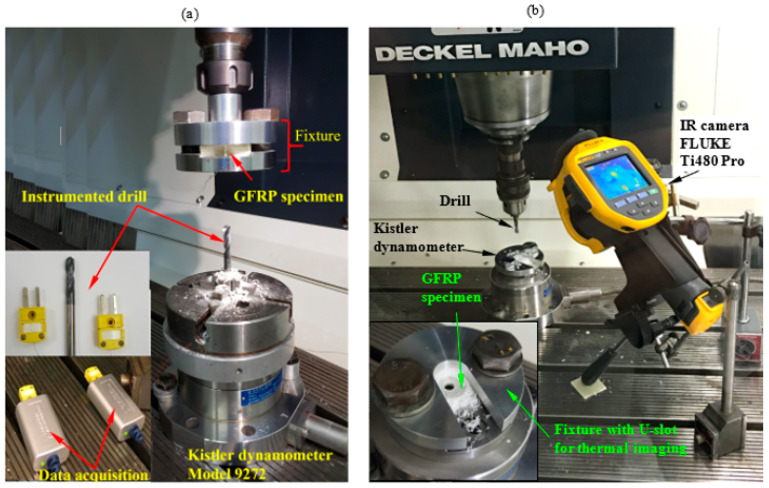
Experimental setup for measuring thrust forces and torque in drilling GFRP composites using CNC milling machine and Kistler dynamometer. The temperature was measured by: (**a**) instrumented drill with two thermocouples and (**b**) IR camera.

Specimens of 36.6 × 36.6 mm were prepared from composite laminates using an abrasive water jet machine. The thrust force and torque are measured by Kistler dynamometer model 9272 connected with PC through multichannel amplifier 5070A and data acquisition (DAQ) type 5697A. The experimental setup with dynamometer-fixture-workpiece assembly is presented in Figure 1. Thrust force and torque data were recorded with a Kistler 9272. For the drilling parameters, a full experimental design is used through spindle speed, feed, the thickness of the sample, and point angles are presented in Table 4.

Through this study, two different techniques were used to measure a temperature. In the first technique, two K-thermocouples were implanted in coolant holes near the drill’s cutting edge. The temperature variation during the drilling process was monitored and recorded using National Instruments LabVIEW Signal Express software. In this method, the instrumented drill was mounted by four independent-jaws chucks, which were fixed on the dynamometer. The specimen was clamped firmly to the machine spindle using a special fixture, as shown in Figure 1a. through the second technique, the specimen was clamped completely on the dynamometer using a special fixture, as shown in Figure 1b. The fixture was designed with a U-slot of 20 mm width to measure the induced temperature in the heat-affected zone (HAZ) using infrared (IR) camera model FLUKE Ti480 Pro. The infrared camera was placed at 260 mm from the hole center and at an angle of 60°, as shown in Figure 1b.

### 2.3. Evolution of Delamination

**Figure 2:**
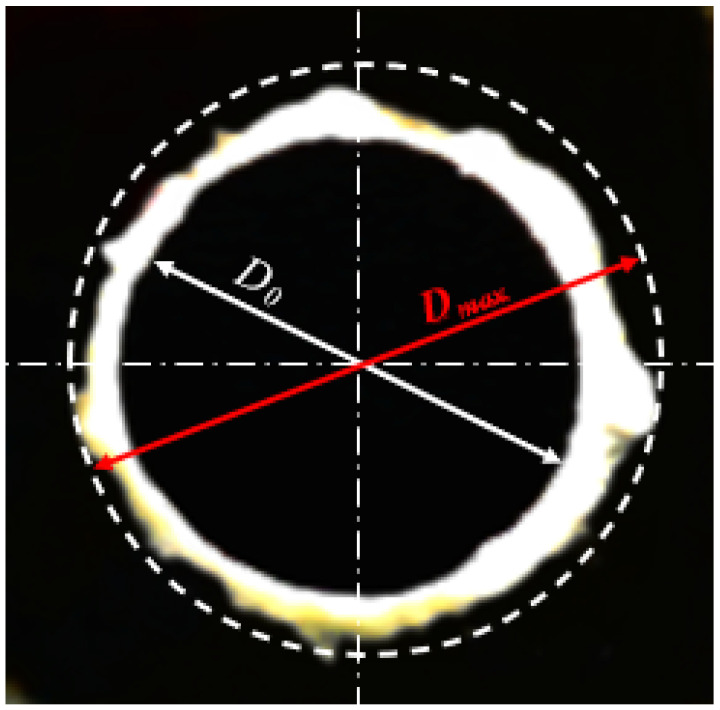
Image indicates the push-out delamination in drilling FRP composites.

The AutoCAD technique was used to characterize the peel-up and push-out surface delamination. More details about this technique are reported earlier by Khashaba [43] and summarized as follows: The technique is appropriate for quasi-transparent composite materials in which the drilled specimen was scanned using high-resolution flatbed color scanner model Epson “V370, 4800 × 9600 dpi”. The transmitted light to the delaminated or damaged zone makes it brighter and can be easily recognized from the undamaged area. The image was explored using CorelDraw software, which facilitates the image treatment and determines the delamination size within 10^–3^ mm. The delamination factor was evaluated by:

(1) $$F_{d}=\frac{D_{max}}{D_{0}}$$

in which $F_{d}$ is the delamination factor, and $D_{max}$ is the maximum delaminated diameter drawn from the centerline of the hole nominal diameter ($D_{0}$ = 6 mm), see Figure 2.

**Figure 3:**
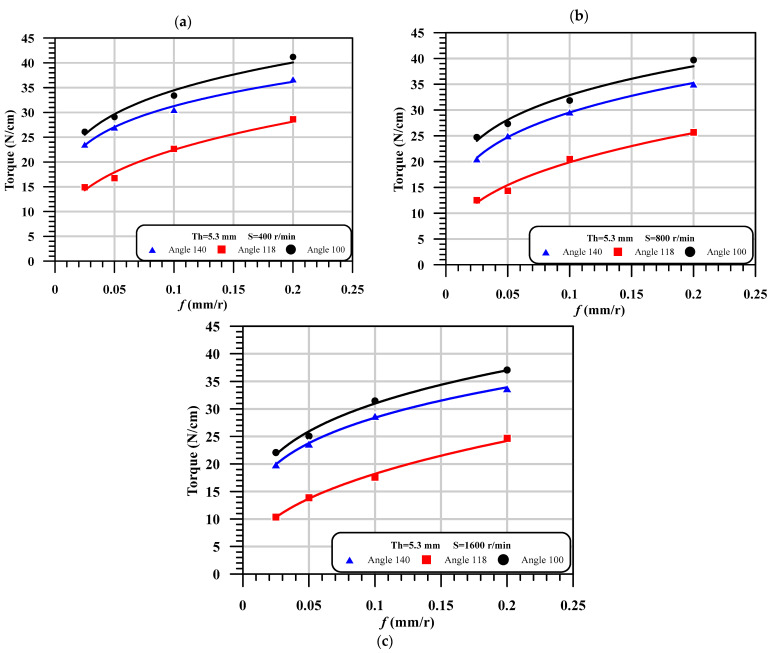
The effect of feed on the delamination factor with different point angles at different speed, (**a**) At thickness of 5.3 mm and speed = 400 r/min; (**b**) At thickness of 5.3 mm and speed = 800 r/min; (**c**) At thickness of 5.3 mm and speed = 1600 r/min.

Influences of drilling parameters, drill point angle, and laminated thickness on the push-out delamination are presented in Table 5, and their quantitative presentation is shown in Figure 3. As shown in this figure, by increasing the point angle of the drill, the push-out delamination increased significantly by increasing the feed. As the case in hand at speed 400 rpm, feed 0.2 mm/r and thickness of 2.6 mm, the variation in drill point angle from 100° to 118° to 140°, and the delamination factor increased from 1.49 to 1.53 to 1.62, respectively. The same observation has been reported [4,41]. It is also noted that the delamination factor is proportional to feed and speed. However, the thickness has a trivial and marginal influence on the delamination factor.

The delamination that occurs in drilling is influenced severely by the mechanical characteristics of the material around the hole. These problems can be prevented by ascertaining the optimum conditions (feed, cutting speed, and material thickness) for a particular machining operation. Therefore, the optimization technique and multivariable regression have been done in the next section to predict the optimum drilling conditions.

## 3. Evolution of Temperature

**Figure 4:**
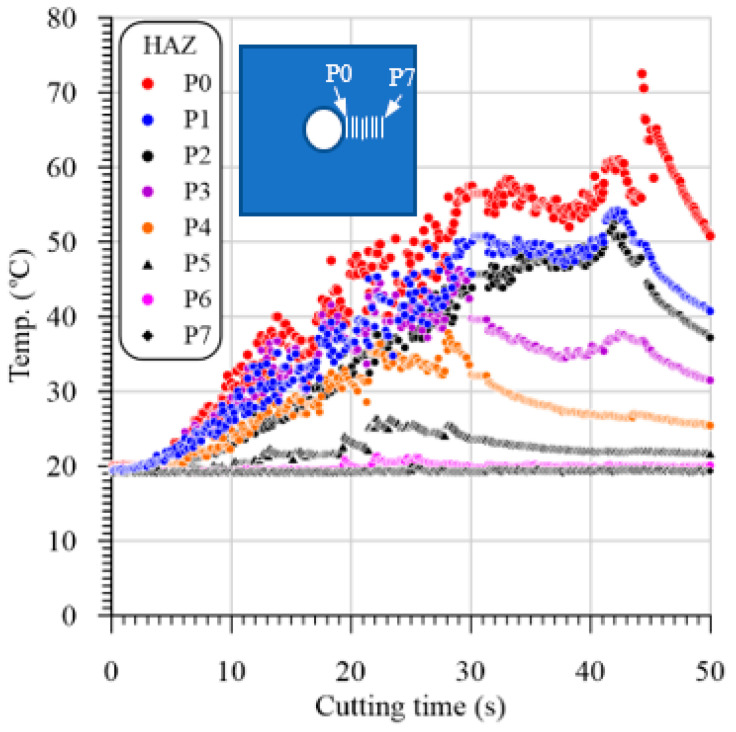
Temperature obtained by IR camera vs. cutting time in drilling GFRP with 5.3 mm thickness at 400 rpm and 0.025 mm/r at different positions using 118° drill point angle.

**Figure 5:**
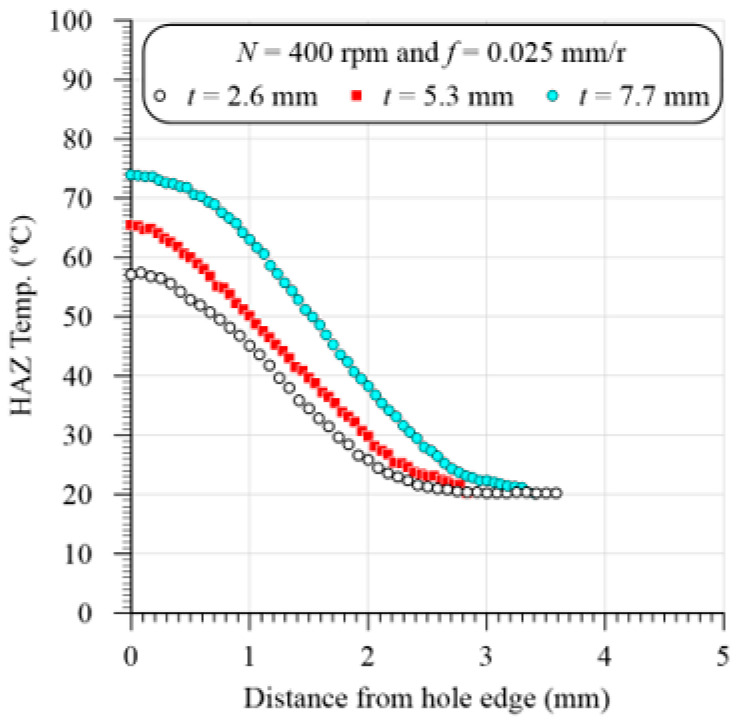
Representative sample of the temperature distribution of HAZ of GFRP specimen with different thicknesses at 400 rpm and 0.025 mm/r.

Temperature rising through the drilling of GFRP composites can result in matrix burnout, debonding of fiber/matrix interface, or even the glass transition of HAZ and hence, severely deteriorate the quality and properties of the composite materials [19]. Figure 4 illustrates a measured temperature using the IR camera versus cutting time in drilling GFRP with 5.3 mm thickness at 400 rpm and 0.025 mm/r at different positions using 118° drill point angle. The heat distribution in the heat-affected zone (HAZ) is obtained using a line of about 5 mm drawn from the hole edge at the middle of the U-slot. Many temperature measurements along the drawn line are recorded, as presented in Figure 5. The distance between temperature measuring lines and with respect to drilled hole edge is about 0.5 mm, which can be demonstrated by comparing Figure 4 and Figure 5. It is obvious that point 0 at zero distance from the hole edge, whereas point 7 (Figure 4) is at a distance of about 3.5 mm (Figure 5). This figure shows samples of the temperature distribution in the HAZ of the GFRP composites with different thicknesses at a speed of 400 rpm and feed of 0.025 mm/r. The results in Figure 5 showed that the HAZ temperature was sharply decreased as it moves away from the hole edge because of the lower thermal conductivity of the GFRP composite laminates. The temperature reached the room temperature of 20 °C after 2.8 mm, 3 mm, and 3.4 mm away from the hole edge of the composite laminates with a thickness of 2.6 mm, 3.5 mm, and 7.7 mm, respectively.

**Figure 6:**
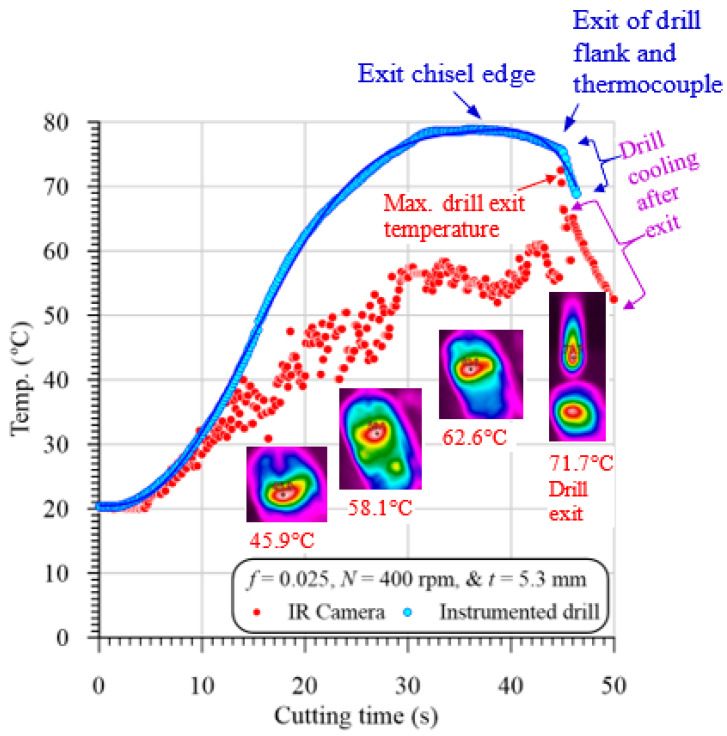
Representative sample of evolution of temperature at hole edge (P0) vs. cutting time in drilling GFRP with 5.3 mm thickness at 400 rpm and 0.025 mm/r using 118° drill point angle.

Figure 6 illustrates a representative sample of the evolution of temperature vs. cutting time in drilling GFRP with 5.3 mm thickness at 400 rpm and 0.025 mm/r. The temperature was measured by both the instrumented drill and the IR camera. It is clear from Figure 6 that the two methods’ measured temperature values at the first 10 s are almost identical. These identical measurements were attributed to that at the drill entry, the chisel edge with zero speed at its center does not cut, but instead, it extrudes the material. Therefore, the camera records the drilling temperature that equal to those measured using the instrumented drill. A similar observation was reported by Xu et al. [17] in drilling CFRP/Ti6Al4V stacks. The higher temperature profile of the measured temperature of the drill flank was at-tributed to the low thermal conductivity of the GFRP composite, which resists heat trans-fer from the too-hole contact interface and thus increases the accumulated drill-flank temperature shown in Figure 6.

On the other hand, the lower temperature profile measured by the IR camera was at-tributed to the thin layers of the chip, which can easily lose some temperature via heat transfer through the flute of the high thermal conductivity drill body. At the drill exit of the work, the IR camera record a sudden increase in the temperature. This because the camera always records the highest temperature in the drilling zone. This result indicates that the drill point temperature (720 °C) is higher than those of the hole edge (620 °C) by about 100 °C, as shown in Figure 6. It is also evident that the maximum temperature recorded by the IR camera is lower than that of the instrumented drill because the IR camera is not directly measuring the tool-work interaction zone during the drilling process. Accordingly, the drill point was partially cooled during the exit of the machined hole. Xu et al. [17] have calibrated the IR camera’s temperature by adding a compensation value equal to the difference between the measurements by the two methods. However, this method is not ac-curate for the following reasons:

- The difference is increased with the specimen thickness, where the drill takes a long time during the exit out of the specimen, and thus loses more heat compared to the thinner one.
- For the same specimen thickness, the difference between the measured temperatures by the two methods is decreased with the increasing feed values, because of the decreasing cutting time, and thus decreasing the measuring time between the two methods.
- In some cases, the hot chips were dropped out of the drill flutes and dispersed on the specimen surface, and thus the measured temperature cannot be calibrated.

Therefore, in the current analysis, the temperature of the instrumented drill was used to construct the different relationships with the cutting variables.

**Figure 7:**
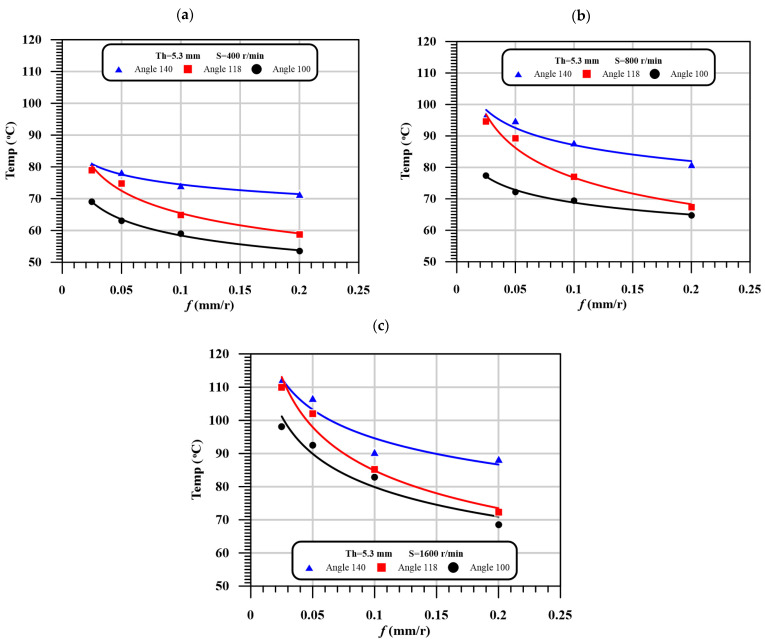
The effect of feed on the heat affected zone with different point angles at different speeds, (**a**) At thickness of 5.3 mm and speed = 400 r/min; (**b**) At thickness of 5.3 mm and speed = 800 r/min; (**c**) At thickness of 5.3 mm and speed = 1600 r/min.

Table 6 shows influences of speed, feed, thickness of laminates, and drill point angle on the HAZ. As presented, by fixing all conditions and increasing the feed, the HAZ decreased significantly within 10% to 25%. However, the speed has the opposite effect on the HAZ. That means the HAZ is increased by increasing the speed or decreasing the feed. It is also noted that, by the increasing drill point angle, HAZ is increased significantly. For example, at 400 rpm speed, 0.025 mm/r feed, and 2.6 mm thickness, HAZ is varied from 50.98, 64.00, to 68.61 °C, as the point angle increased from 100°, 118°, to 140°, respectively. The influence of thickness is the same as the influence of drill point angle on the HAZ. Thus, by increasing the thickness of laminates, the time of machining is increased, and hence the amount of heat generated during cutting increased. The qualitative presentation of Table 6 is presented in Figure 7 at a specific thickness.

## 4. Mechanical Results

This section presents the response of the GFRP laminated structure during the drilling process with different drill angles. The evaluation of critical thrust force, torque as a mechanical response, delamination as failure response, and heat-affected zone as a thermal response are studied under drilling (speed and feed) and geometrical (thickness of laminates and drill angle) parameters. The variation of machining response concerning machining time is also discussed in detail. The optimum drilling conditions and statistical analysis for drilling GFRP laminated with different thicknesses using different drilling angles are studied in the next section.

### 4.1. Evolution of Thrust Force

At the beginning of the drilling process, the thickness of the laminate can sustain the axial force. Though, this thickness cannot withstand this load when the drill approaches the exit. Therefore, the thrust force applied on the uncut thickness exceeds the inter-ply shear stress and then causes severe damage known as the push-out delamination. The critical thrust force is the main parameter responsible for delamination, especially for push-down delamination [42]. The variation of the critical thrust force vs. the drilling and geometrical parameters is presented in Table 7. As shown, by increasing the feed of drilling and fixing the other parameters, the thrust force increased significantly. Therefore, the thrust force can be presented as a proportional function of feed. Some researchers observed similar qualitative behavior for different composite systems and drill geometries [23,29,44,45].

By comparing the influence of speed vs. feed on the thrust force, it is found that the speed effect is more trivial to the feed. For example, at Th = 2.6 mm, angle 118°, by doubling the feed from 0.025 to 0.0.05 mm/r at speed 400 rpm, the thrust force increased by 35% by doubling the speed from 400 to 800 rpm at 0.025 mm/r, the thrust force unchanged. That may be due to the heat-affected zone that will be discussed later.

It is observed that point angle significantly affects the critical thrust force, especially for higher feed and speed. For example, at Th = 5.3 mm, speed 1600 rpm and feed 0.2 mm/r, the increasing of point angle from 100°, 118°, to 140° tends to increase the critical thrust force from 85.73, 94.74 to 132.55 N, respectively, with the percentage of 110 % and 154% relative to the smallest angle. The thickness has an inconsistent influence on the thrust force.

**Figure 8:**
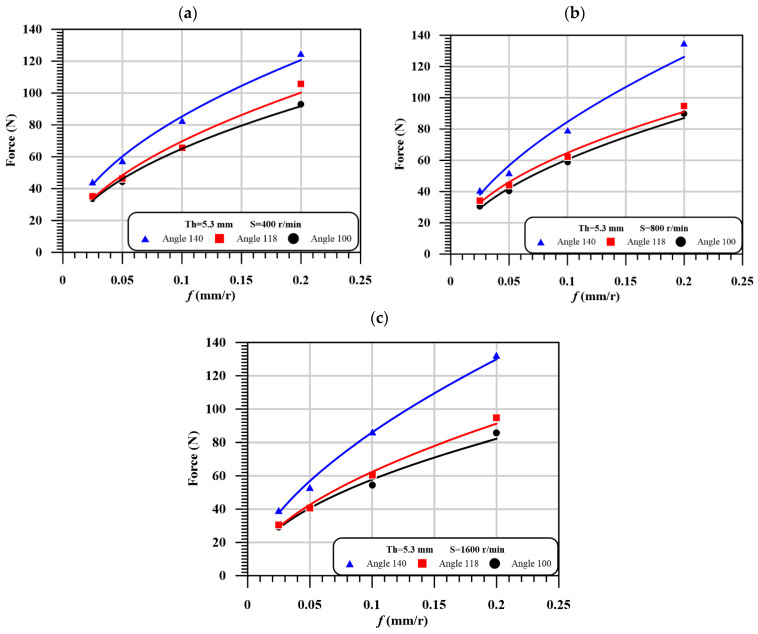
The influence of feed on the critical thrust force with different point angles at different speeds, (**a**) At thickness of 5.3 mm and speed = 400 r/min; (**b**) At thickness of 5.3 mm and speed = 800 r/min; (**c**) At thickness of 5.3 mm and speed = 1600 r/min.

As shown in Table 7, by increasing the thickness from 2.6 mm to 5.3 mm, the thrust force increased by 10% to 15%. This increase is due to the increase of laminated rigidity due to increasing the thickness. However, increasing the thickness of laminated from 5.3 mm to 7.7 mm, causes a reduction in critical thrust force. This reduction is due to the reduction of rigidity caused by increasing the heat-affected zone and drilling time. A qualitative presentation of Table 7, which represents the impact of speed, feed, and point angle on the critical thrust force at a laminated thickness of 5.3 mm, is illustrated in Figure 8.

### 4.2. Evolution of Torque

**Figure 9:**
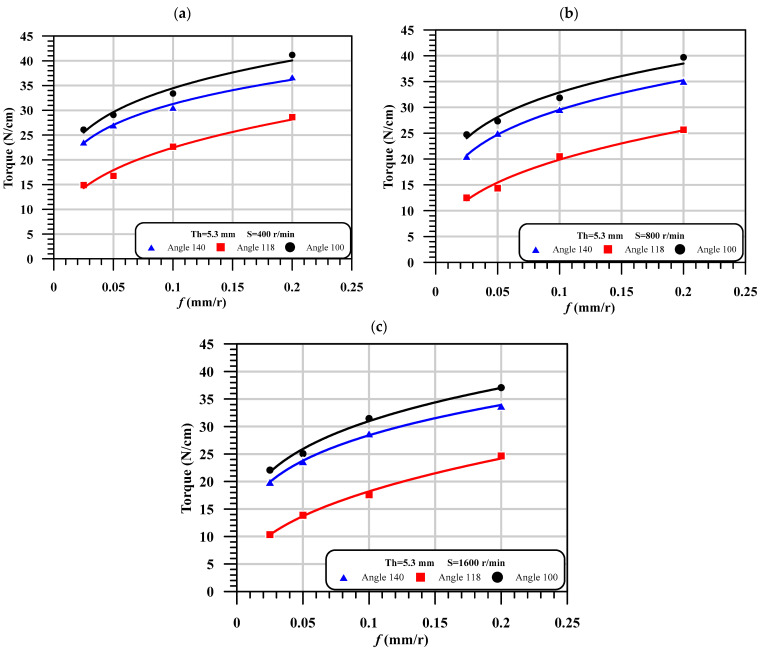
The effect of feed on the torque with different point angles at different speeds, (**a**) At thickness of 5.3 mm and Speed = 400. r/min; (**b**) At thickness of 5.3 mm and speed = 800 r/min; (**c**) At thickness of 5.3 mm and speed = 1600 r/min.

The torque evaluated during the drilling process is considered as the 2nd parameter response after the critical thrust force. The torque is measured by Kistler dynamometer model 9272 that connected with PC through multichannel amplifier 5070A and data acquisition (DAQ) type 5697A. The variation of the torque with machining and the geometrical parameter is presented in Table 8. The qualitative representation of Table 8 at thickness 5.3 mm is illustrated in Figure 9. It is noted in this figure that the torque is proportional with the feed, which means that the increase of torque is caused by increasing the input feed. It is observed that the drill point angle has different effects on the torque. As shown, the highest torque is observed in the case of the smallest point angle at 100°. However, the lowest torque is noticed at the middle point angle 118°. The induced torque at point angle 140° is between the torque of point angle 100° and the torque of point angle 118°. It can be observed from Table 6 that the influence of thickness on the torque is vice versa influenced by the point angle of the drill. As seen, as the thickness increased from 2.6 to 5.3 mm, the torque increased by ~40%; however, increasing the thickness from 5.3 to 7.7 mm, the torque decreased, especially at a point angle of 140°.

### 4.3. Machining Responses vs. Machining Time

**Figure 10:**
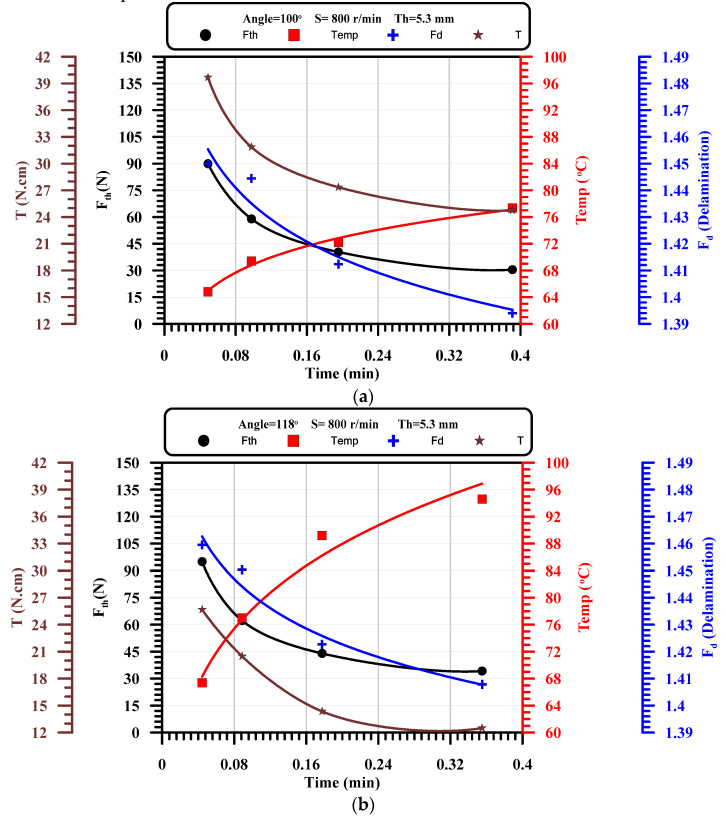

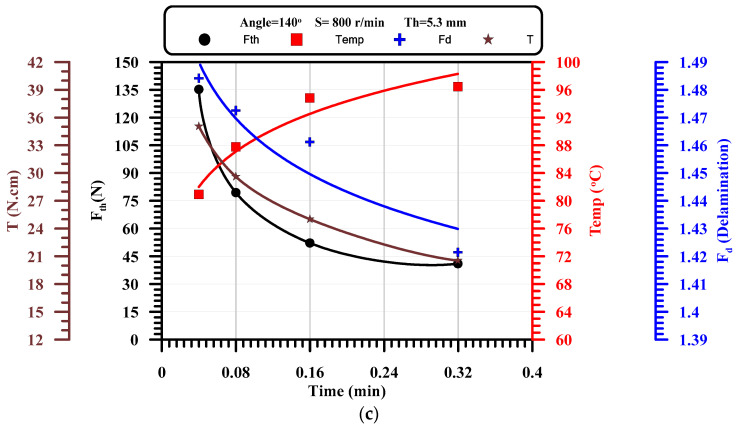
The effect of machining time on thrust force, torque, delamination, and temperature at constant speed and thickness and different point angles, (**a**) At thickness of 5.3 mm and speed = 800 r/min, Angle = 100°; (**b**) At thickness of 5.3 mm and speed = 800 r/min, Angle = 118°; (**c**) At thickness of 5.3 mm and speed = 800 r/min, Angle = 140°.

The drilling process is a very complicated problem that can be analyzed element by element, which means the influence of feed on delamination or feed on thrust force because the thrust force as the output will affect the delamination. Therefore, the current section tries to combine the coupling effect of drilling responses with the machining time, which is a function of the input parameters (thickness, drill point angle, speed, and feed). Figure 10 presents the coupling influences of input drilling parameters collected in machining time to output drilling parameters (thrust force, torque, delamination, and temperature). As shown from these figures, the thrust force and delamination have the same behaviors rather than the temperature with the variation of drilling time, which assures that the delamination is dependent proportionally on the thrust force and inversely with the temperature that may lead to the softening. Therefore, the thrust force and temperature have a coupling effect on the delamination ratio, which will be investigated statistically in the next section. From Figure 10, it can be concluded that, by increasing drilling time, the temperature of drill and chip increased, and the thrust forced decreased in exponential forms.

## 5. Statistical Analysis

In the last few years, considerable attention has been paid to use multiple regression models for correlating machinability parameters with machining conditions in drilling fiber-reinforced composites [46,47,48,49,50]. The design experiment methods have been used extensively in investigating the significance of cutting condition factors on the delamination of fiber composites during the drilling process. Box Behnken design with a simulation annealing algorithm was used for the development of the regression model to control the thrust force and delamination in drilling of Graphene oxide/CFRP nanocomposites by Kumar et al. [51]. Di Benedetto et al. [52] employed artificial neural networks and design experiment methods for developing a prediction model of energy absorption capability of thermoplastic composites. Much research combined between design experiment methods and neural networks to develop prediction models [53,54].

As outputs of the drilling operation, thrust force, torque, and temperature were measured during the experiment conducting. In the present study, a factorial design was used to identify the main effects of three factors named feed, spindle speed, and workpiece thickness on the machinability responses mentioned above. The machining properties were measured according to design of experiments for actual independent drilling process variables with their levels illustrated in Table 4.

### 5.1. Statistical Results

The primary objective for employing ANOVA was to investigate the significance of machining parameters affecting the machinability properties including thrust force, torque, cutting temperature, and delamination factor. The ANOVA results are summarized in Table 9. The contribution percentage of each parameter on the total variation indicates its effect on the measured properties. The significance of the effect of the machining parameters on the machinability of the GFRP composite can be measured by the *p*-value. For most experimental work, the *p*-value less than 0.05 indicates the significance of the related factor for the response. Accordingly, all machining parameters have a significant effect on the measured temperature as shown in Table 9. The largest contribution is of the laminate thickness (29.00%), followed by speed and feed (29.00%, and 15.10%, respectively), ended by the lowest contribution of the drill point angle (11.85%).

The contribution of the feed on measured thrust force is about 84.63%, which is higher than those of drill point angle (7.37%), followed by the laminate thickness (1.94%). However, the effect of laminate thickness is higher than the cutting speed (0.38%), which is agreed with Figure 6. The lower contribution of the speed was attributed to the indirect effect of increasing the temperature accompanied by decreasing stiffness of GFRP specimen on the measured force. Regarding measured torque, it is primarily affected by feed (44.93%), followed by the drill point angle (33.37%), then the thickness (15.47%), while the lowest effect is of the speed.

**Figure 11:**
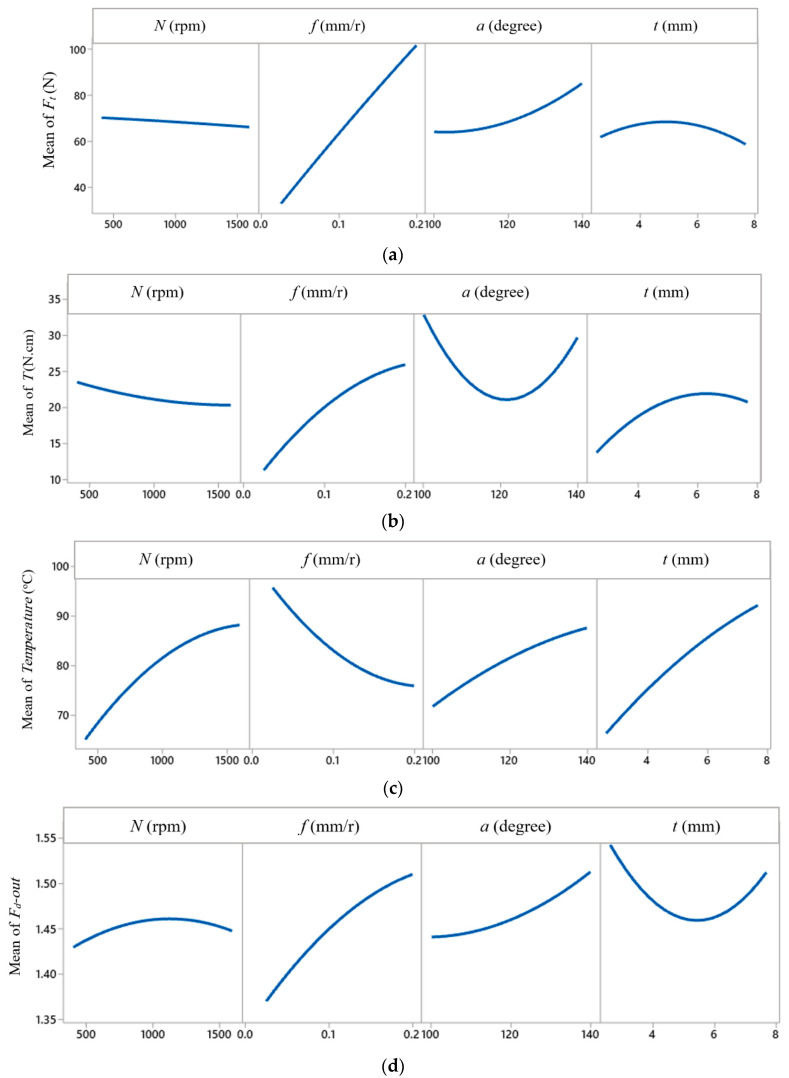
Main effect plots: (**a**) Ft (N), (**b**) T (N.cm), (**c**) temperature (°C), and (**d**) Fd-out.

Since the drilling parameters are considered at multiple levels, quadratic mathematical models based on response surface methodology are developed to predict machinability properties. The equation of a predicted response (*Y*) can be expressed as follows:

(2) $$Y=\beta_{0}+\beta_{1}N+\beta_{2}f+\beta_{3}a+\beta_{4}t+\beta_{11}N^{2}+\beta_{22}f^{2}+\beta_{33}a^{2}+\beta_{44}t^{2}+\beta_{12}Nf+\beta_{13}Na+\beta_{14}Nt+\beta_{23}fa+\beta_{24}ft+\beta_{34}at$$

where *Y* is the response, *N*, *f*, *a*, and *t* are the design factors, and the $\beta^{\prime }s$ are the coefficients of variation resources of the prediction response model, which are listed in Table 10 beside the regression coefficient (R^2^) value of each estimated model. The higher values of R^2^ indicate that the predicted machinability properties have good agreement with the experimental results. The regression models were used to generate the response surface plots for all machinability properties. To assist in the interpretation of this experimental study, Figure 11 presents the main effect plots for the variation of thrust force, torque, temperature, and delamination factor concerning drilling parameters. Those plots summarize the analysis explained above, where the main effect plots identify influencing the response as well as the direction of the relationship between factors and response.

**Figure 12:**
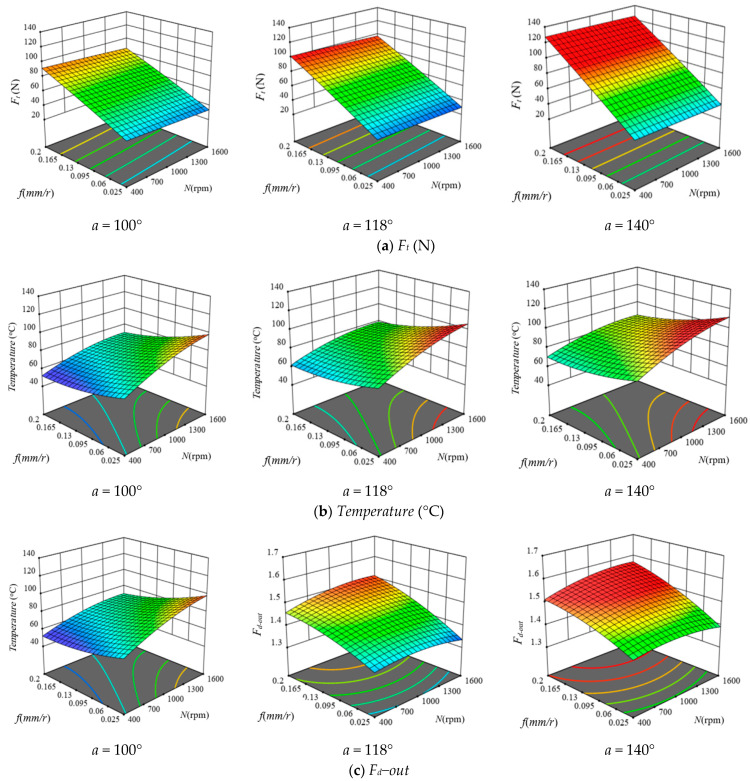
Response surface plots of drilling parameters effect on the machinability properties of GFRP composite with thickness of 5.3 mm: (**a**) Ft, (**b**) temperature, and (**c**) Fd−out.

Figure 12 illustrates 3D surface and contour plots of machinability responses versus different drilling parameters of GFRP composite with a thickness of 5.3 mm, as a representative sample. These plots can easily indicate the critical conditions for the predicted machinability properties. For example, at the feed of 0.2 mm/r, the critical thrust force was observed at speed of 400 rpm, as shown in Figure 12a. Similarly, the critical temperature was observed at the feed of 0.025 mm/r and speed of 1600 rpm using a drill with a point angle of 140°, as shown in Figure 12b. Response surface analysis through Figure 12c indicates the critical push−out delamination factor was observed at the feed of 0.2 mm/r using a drill point angle of 140°. Likewise, optimum conditions can be inferred.

### 5.2. Optimization of Delamination Factor

**Figure 13:**
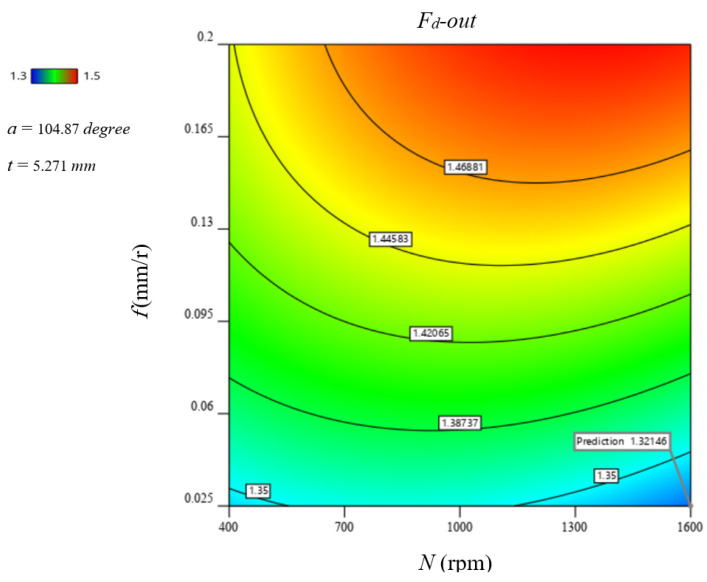
Contour plot of delamination−exit ratio at optimal parameters N = 1600 rpm and f = 0.025 mm/r.

The vital portion of this experimental work is to determine the optimal drilling parameters where minimum delamination during drilling in GFRP laminate. Response surface regression modeling is used to optimize the drilling operation responses identifying the optimal drilling conditions. Concerning all drilling operation responses, the analysis is performed considering that a smaller value is better for optimization. Applying the numerical optimization function of Design-Expert software, an optimization solution of the drilling parameter is obtained. The response surface and contour plot at optimal drilling parameters for minimum delamination ratio are shown in Figure 13. In a range of this study, to produce a quality hole with minimum push-exit delamination, the optimal drilling parameters are the feed of 0.025 mm/r, and the speed of 1600 rpm, and the use of a smaller drill point angle with a material thickness of 5.124 mm as shown in Figure 13, ignoring other drilling responses. This combination may produce minimum push-exit delamination but is associated with maximum temperature, as shown in the part of the plot dedicated for temperature in Figure 13.

The developed model and results obtained should be validated at optimum and random points. For this purpose, estimated results according to regression models listed in Table 10 and data obtained from the experiments are shown in Table 11 for delamination-exit. It is evident to observe that the inferential and the experimental results are close to each other. Regarding reliability, statistical analysis errors should be limited to 20% [4]

## 6. Conclusions

Impacts of machining parameters, such as cutting speed, feed, point angle of the drill, and thickness of specimen on thermal and mechanical responses of GFRP laminates have been investigated experimentally and statistically. The heat−affected zone (HAZ) and drill point temperature are evaluated and studied by thermal infrared camera and thermocouples. Drilling studies are carried out using a CNC machine under dry cutting conditions by 6 mm diameter twist drill of cemented carbide drill with different point angles. The main results from this study are:

- By increasing the feed of drilling and fixing the other parameters, the thrust force increased significantly. Therefore, the thrust force can be presented as a proportional function of feed.
- The temperature of the HAZ was sharply decreased as it moved away from the hole edge as a result of the lower thermal conductivity of the GFRP composite laminates.
- By comparing the influence of speed vs. feed on the thrust force, it is found that the speed effect is more trivial with respect to the feed.
- It is observed that the point angle has a significant effect on the critical thrust force, especially for higher feed and speed.
- By increasing the point angle of the drill, the push−out delamination increased significantly by increasing the feed.
- The thrust force and delamination have the same behaviors rather than the temperature with the variation of drilling time, which assure that the delamination is dependent proportionally on the thrust force and inversely with the temperature that may lead to the softening.
- Accordingly, all machining parameters have a significant effect on the measured temperature, the largest contribution is of the laminate thickness (33.14%), followed by speed and feed (29.00% and 15.10%, respectively), ended by the lowest contribution of the drill point angle (11.85%).

## Figures and Tables

**Table 1:** Mechanical properties of woven GFRP composites.

Property	Value	Dimension	Standard Deviation
Poisson’s ratio (*υ*_12_ = υ_21_)	0.295	—	0.015
Young’s modulus (*E*_11_ = *E*_22_)	16.05	GPa	0.116
Tensile Strength	203.86	MPa	4.215

**Table 2:** The constituent materials of the cemented carbide drills [23].

Material Grade	ISO Code	WC	Co	Grain Size (µm)	Density (g/cm^3^)	Hardness (HRA)	Transverse Rupture Strength (MPa)	K_IC_ (MPa·m^1/2^)
K200	K20~K40	90%	10%	0.5~0.8	14.4	91.3	3920	10.5

**Table 3:** Geometries of the cemented carbide drills.

D (mm)	Flute Length (mm)	Overall Length (mm)	Helix Angle	Rake Angle	Clearance Angle	Point Angles	Chisel Edge Length (mm)
6	28	66	30°	30°	12°	100°/118°/140°	0.3

**Table 4:** Levels of the variables used in the experiment.

Factors	Unit				
Spindle speed, N	rpm	400 (7.5 m/min)	800 (15 m/min)	1600 (30 m/min)	–
Feed, f	mm/r	0.025	0.05	0.1	0.2
Thickness of sample, t	mm	2.6	5.3	7.7	–
Point angle	deg	100°	118°	140°	–

**Table 5:** The variation of delamination with speed, feed, thickness, and point angle.

Speed rpm	Feed (mm/r)	Th = 2.6 mm			Th = 5.3 mm			Th = 7.7 mm		
		Angle 100°	Angle 118°	Angle 140°	Angle 100°	Angle 118°	Angle 140°	Angle 100°	Angle 118°	Angle 140°
400	0.025	1.39	1.40	1.51	1.38	1.38	1.39	1.41	1.40	1.42
	0.05	1.42	1.44	1.56	1.39	1.40	1.42	1.44	1.42	1.47
	0.1	1.45	1.47	1.58	1.39	1.40	1.46	1.50	1.47	1.51
	0.2	1.49	1.53	1.62	1.43	1.43	1.50	1.57	1.55	1.59
800	0.025	1.42	1.45	1.55	1.39	1.41	1.42	1.39	1.40	1.42
	0.05	1.49	1.50	1.58	1.41	1.42	1.46	1.42	1.42	1.46
	0.1	1.49	1.53	1.62	1.44	1.45	1.47	1.47	1.46	1.55
	0.2	1.58	1.58	1.68	1.45	1.46	1.48	1.54	1.52	1.62
1600	0.025	1.35	1.36	1.48	1.32	1.34	1.34	1.37	1.45	1.44
	0.05	1.41	1.43	1.53	1.40	1.41	1.44	1.40	1.47	1.49
	0.1	1.48	1.50	1.59	1.43	1.43	1.44	1.43	1.51	1.57
	0.2	1.60	1.65	1.65	1.50	1.51	1.53	1.52	1.57	1.63

**Table 6:** The variation of HAZ with speed, feed, thickness, and point angle.

Speed rpm	Feed (mm/r)	Th = 2.6 mm			Th = 5.3 mm			Th = 7.7 mm		
		Angle 100°	Angle 118°	Angle 140°	Angle 100°	Angle 118°	Angle 140°	Angle 100°	Angle 118°	Angle 140°
400	0.025	50.98	64.00	68.61	69.09	78.89	80.55	72.82	83.10	87.69
	0.05	49.17	60.66	63.92	63.06	74.77	78.33	62.70	79.97	85.82
	0.1	46.24	57.20	59.27	59.02	64.89	74.15	58.79	70.62	82.60
	0.2	45.88	50.78	58.38	53.53	58.79	71.42	53.95	65.54	78.19
800	0.025	59.78	76.74	78.45	77.35	94.60	96.45	92.03	103.30	104.25
	0.05	57.16	72.37	75.82	72.17	89.20	94.82	85.33	96.67	99.35
	0.1	56.11	67.86	73.78	69.40	76.99	87.78	78.19	85.43	93.02
	0.2	55.57	58.19	71.91	64.80	67.37	80.91	69.44	75.71	80.41
1600	0.025	64.79	81.17	83.89	98.06	109.91	111.89	120.00	127.84	129.38
	0.05	59.06	74.81	81.73	92.54	102.07	106.73	108.37	116.55	125.70
	0.1	58.39	67.08	78.76	82.92	85.18	90.37	95.00	101.57	110.73
	0.2	56.17	60.46	77.33	68.57	72.31	88.38	80.00	86.45	95.09

**Table 7:** The variation of critical thrust force with speed, feed, thickness, and point angle.

Speed rpm	Feed (mm/r)	Th = 2.6 mm			Th = 5.3 mm			Th = 7.7 mm		
		Angle 100°	Angle 118°	Angle 140°	Angle 100°	Angle 118°	Angle 140°	Angle 100°	Angle 118°	Angle 140°
400	0.025	29.94	30.47	30.99	33.56	35.32	44.25	31.68	27.31	31.46
	0.05	38.01	40.72	47.04	44.20	46.33	57.55	41.47	36.60	43.94
	0.1	54.26	56.20	74.24	65.02	65.74	82.81	59.35	53.05	62.48
	0.2	83.70	95.72	126.12	93.03	105.74	124.93	88.01	85.34	108.74
800	0.025	29.46	35.32	30.99	30.44	34.20	41.01	30.98	30.41	27.59
	0.05	34.66	46.33	47.04	40.34	44.00	52.19	42.95	40.62	43.65
	0.1	52.07	65.74	74.24	58.93	62.26	79.46	60.07	60.39	69.49
	0.2	79.06	105.74	126.12	89.91	94.92	135.25	89.28	94.74	119.79
1600	0.025	26.54	26.59	30.49	29.28	30.41	39.37	27.88	24.52	25.49
	0.05	35.01	34.58	45.95	40.18	40.62	53.07	37.72	32.60	36.88
	0.1	47.09	51.09	76.42	54.46	60.39	86.41	50.11	49.18	56.28
	0.2	77.51	90.65	126.50	85.73	94.74	132.55	81.50	82.28	110.53

**Table 8:** The variation of torque with speed, feed, thickness, and point angle.

Speed rpm	Feed (mm/r)	Th = 2.6 mm			Th = 5.3 mm			Th = 7.7 mm		
		Angle 100°	Angle 118°	Angle 140°	Angle 100°	Angle 118°	Angle 140°	Angle 100°	Angle 118°	Angle 140°
400	0.025	17.76	9.27	16.53	26.10	14.91	23.90	22.75	13.91	11.03
	0.05	21.70	11.04	18.63	29.08	16.75	26.93	28.81	16.74	15.06
	0.1	26.33	14.97	22.38	33.39	22.64	30.11	35.07	21.22	23.96
	0.2	30.04	20.11	28.22	41.19	28.62	35.39	43.74	27.11	37.79
800	0.025	16.13	7.89	13.20	24.74	12.50	20.54	21.09	11.30	18.49
	0.05	20.35	10.43	14.99	27.34	14.35	25.01	25.12	14.51	21.24
	0.1	24.33	14.13	22.74	31.85	20.48	29.61	33.17	19.37	30.10
	0.2	28.58	19.11	27.42	39.69	25.67	35.05	41.56	24.83	37.16
1600	0.025	14.83	7.25	12.70	22.08	10.35	19.91	19.91	10.11	15.00
	0.05	19.19	9.26	15.45	25.06	13.87	23.67	24.21	12.98	18.36
	0.1	23.10	12.70	22.13	31.45	17.58	28.72	30.22	17.95	29.80
	0.2	27.10	19.06	24.62	37.07	24.66	33.74	39.64	23.13	35.12

**Table 9:** ANOVA results with contribution of control factors effect on machinability responses.

Source of Variation	DF	Ft	*p*-Value	T (N·cm)	*p*-Value	Temp	*p*-Value	Fd-Out	*p*-Value
N (rpm)	2	0.38%	0.040	2.51%	0.000	29.00%	0.000	1.45%	0.018
f (mm/r)	3	84.63%	0.000	44.93%	0.000	15.10%	0.000	45.95%	0.000
a (degree)	2	7.37%	0.000	33.77%	0.000	11.85%	0.000	15.47%	0.000
t (mm)	2	1.94%	0.000	15.47%	0.000	33.14%	0.000	20.10%	0.000
Error	28	5.68%		3.32%		10.91%		17.03%	
Total	35	100.00%		100.00%		100%		100.00%	

**Table 10:** Quadratic regression model for machinability responses.

Coeff.	Coeff. Value of *Y* Response				Coeff.	Coeff. Value of *Y* Response			
	Ft	T	Temp.	Fd-Out		Ft	T	Temp.	Fd-Out
B0	190.8965	364.478	−95.7782	1.856842	B44	−1.25229	−0.5976	−0.35499	0.010372
B1	−0.01098	−0.00722	0.041555	6.37 × 10^−5^	B12	0.001026	0.000562	−0.06571	0.000341
B2	−155.18	126.3083	−108.724	0.93023	B13	8.22 × 10^−5^	1.1 × 10^−5^	−6.2 × 10^−5^	2.31 × 10^−7^
B3	−3.51025	−6.14827	1.652451	−0.0071	B14	−0.00027	−0.00023	0.003847	8.56 × 10^−7^
B4	20.15451	7.70022	9.057626	−0.07763	B23	4.875427	−0.01647	0.23058	0.00114
B11	−5.2 × 10^−7^	2.19 × 10^−6^	−1.4 × 10^−5^	−6 × 10^−8^	B24	−0.99698	6.337143	−17.8264	−0.0036
B22	−144.372	−328.04	557.2154	−2.61701	B34	−0.06242	−0.00542	−0.01807	−0.0003
B33	0.015528	0.025368	−0.00469	4.2 × 10^−5^	R2	0.9851	0.9795	0.9689	0.8663

**Table 11:** Verification of the results for Fd−out.

N (rpm)	f (mm/r)	a (Degree)	t (mm)	Status	Fd-Out Exp.	Fd-Out Pred.	Fd-Out Error (%)
1600	0.025	100	5.3	Optimal	1.3215	1.3200	0.114
800	0.05	118	2.6	Random	1.4977	1.4763	1.424
400	0.2	140	7.7	Random	1.5936	1.5485	2.829
400	0.05	140	2.6	Random	1.5572	1.5291	1.806
800	0.025	100	5.3	Random	1.3940	1.3544	2.842
400	0.2	100	5.3	Random	1.4257	1.4433	1.232

## Data Availability

All data avaliable on request.
